# Collaborative Ambulatory Orthopaedic Care in Patients with Hip and Knee Osteoarthritis: A Retrospective Comparative Cohort Study on Health Utilisation and Economic Outcomes

**DOI:** 10.5334/ijic.6442

**Published:** 2023-06-02

**Authors:** Angelina Müller, Sebastian Gruhn, Olga A. Sawicki, Anastasiya Glushan, Claudia Witte, Renate Klaaßen-Mielke, Burkhard Lembeck, Martin Beyer, Ferdinand M. Gerlach, Wolfgang Greiner, Kateryna Karimova

**Affiliations:** 1 Institute of General Practice, Goethe University, Theodor-Stern-Kai 7, Frankfurt, 60590, DE; 2 Health Economics and Health Care Management, Bielefeld University, P.O. Box 10 01 31, 33501, Bielefeld, DE; 3 aQua, Institute for Applied Quality Improvement and Research in Health Care, 37073 Goettingen, DE; 4 Department of Medical Informatics, Biometry and Epidemiology, Ruhr-University Bochum, DE; 5 Joint Practice for Orthopaedic and Trauma Dr. Lembeck und Dr. Pampel, Hindenburgstr. 7/1, 73760, Ostfildern-Nellingen, DE

**Keywords:** osteoarthritis, collaborative healthcare, health service utilisation, multivariable analysis, economic outcomes

## Abstract

**Objective:**

To evaluate a novel healthcare programme for the treatment of patients with hip and knee osteoarthritis in southern Germany in terms of clinical and health economic outcomes. The study is based on claims data from 2014 to 2017.

**Methods:**

We conducted a retrospective comparative cohort study of 9768 patients with hip and knee osteoarthritis, of whom 9231 were enrolled in a collaborative ambulatory orthopaedic care programme (intervention group), and 537 patients received usual orthopaedic care (control group). Key features of the programme are coordinated care, morbidity-adapted reimbursement and extended consultation times. Multivariable analysis was performed to determine effects on health utilisation outcomes. The economic analysis considered annual costs per patient from a healthcare payer perspective, stratified by healthcare service sector. Besides multivariable regression analyses, bootstrapping was used to estimate confidence intervals for predicted mean costs by group.

**Results:**

Musculoskeletal-disease-related hospitalisation was much less likely among intervention group patients than control group patients [odds ratio (OR): 0.079; 95% CI: 0.062–0.099]. The number of physiotherapy prescriptions per patient was significantly lower in the intervention group (RR: 0.814; 95% CI: 0.721–0.919), while the likelihood of participation in exercise programmes over one year was significantly higher (OR: 3.126; 95% CI: 1.604–6.094). Enrolment in the programme was associated with significantly higher ambulatory costs (€1048 vs. €925), but costs for inpatient care, including hospital stays, were significantly lower (€1003 vs. €1497 and €928 vs. €1300 respectively). Overall annual cost-savings were €195 per patient.

**Conclusions:**

Collaborative ambulatory orthopaedic care was associated with reduced hospitalisation in patients with hip and knee osteoarthritis. Health costs for programme participants were lower overall, despite higher costs for ambulatory care.

## Background

Epidemiological data on the incidence and prevalence of osteoarthritis (OA) are sparse in Germany [1] and vary depending on how OA is defined [2]. The number of self-reported diagnoses is higher than that of clinical diagnoses [3]. Experts estimated 5 million people in Germany were living with symptomatic OA in 2016 [2]. In 2010, representative population-based surveys showed that the 12-month prevalence of OA is highest in women (46.9%) and men (30.5%) over 65 years of age. Recent studies based on German claims data indicate prevalence rates from 15.3% to 31.0%, with the highest rates occurring among people aged 80–89 years [4]. Since OA is characterised by constant pain and physical and mental impairment, it greatly affects patients’ quality of life [5,6,7]. It also imposes a substantial economic burden on society, with healthcare costs two to four times higher in OA patients than non-OA patients [5,6,8]. In the U.S., OA was responsible for all-cause direct costs of $359.5 billion and all-cause total costs of $550.2 billion, whereby disease-attributable costs were $80.6 billion and $154.8 billion respectively [9]. In Germany, OA was responsible for health care costs of €8.71 billion in 2015 [10], or 2.6% of total annual health care costs. Since this chronic condition continues to impose a significant burden on patients and healthcare systems, it is important to manage ambulatory care more effectively.

According to Osteoarthritis Research Society International recommendations [11], the initial focus of ambulatory care should be on self-help and patient-driven treatments. While patient empowerment is considered a key component [12] of chronic care for patients with OA, little is known about the individual components of self-management and education programmes [11].

Collaborative ambulatory orthopaedic care, referred to here as the orthopaedic care programme, is a stepped healthcare model that involves coordinated general practitioner (GP) referrals to orthopaedists, and continuous care. Key components are extended consultation times (whereby consultations are sociodemographic background-adapted and motivational), strengthened non-pharmacological therapies, and evidence-based patient information. Participation in GP-centred care, which is a precondition for inclusion in the orthopaedic care programme, is a structured and evidence-based programme for patients with chronic diseases. The success of GP-centred care appears to result largely from the financial incentivisation of gatekeeping [13]. Orthopaedic specialists that wish to participate in the programme must follow the stepped-care approach, include elements of managed care, and improve collaboration between GPs and specialists [14]. Such specialist programmes as the cardiology care programme have been shown to support chronic care and reduce hospitalisation risk in selected patients in southern Germany [14]. However, the implementation of strong and efficient health care programmes for OA has so far proved challenging [12]. This study evaluates the first OA healthcare programme in Germany designed to strengthen cooperation between primary care and specialist care and improve patient-centred consultations in the field of orthopaedic ambulatory care.

## Methods

### Study design and setting

We conducted a retrospective comparative cohort study based on routinely available claims data from the statutory health insurance fund ‘Allgemeine Ortskrankenkasse’ (AOK) in the federal state of Baden-Wuerttemberg, Germany, from 2014 to 2017. During the study period, AOK provided health insurance to about 5.1 million of Baden-Wuerttemberg’s 11.1 million inhabitants [15], making it the largest health insurance fund in the state [16]. In 2016, about 550 orthopaedists and 350,000 insured persons participated in the orthopaedic care programme [17].

### Participants and data availability

Data selection was based on insurance status and diagnosis. Inclusion criteria for patients were a diagnosis of hip OA (ICD-Code M16.-), knee OA (ICD-Code M17.-), or both, uninterrupted health insurance, residence in Baden-Wurttemberg, and aged over 18 years.

Persons diagnosed with OA between September 2015 and September 2016 were included, while those diagnosed between 2014 and August 2015 were not. Empty fields in the dataset were treated as missing values. Outcomes were assessed 365 days after inclusion. In 2015, comorbidities and baseline characteristics of patients were recorded for a pre-observation period.

The intervention group consisted of patients enrolled in the orthopaedic programme that had consulted an orthopaedist participating in the programme at least once. The control group received usual care, which in our case meant patients were not enrolled in either the orthopaedic or the GP-centred care programme and had seen an orthopaedist that was not participating in the programme at least once.

**Figure 1:**
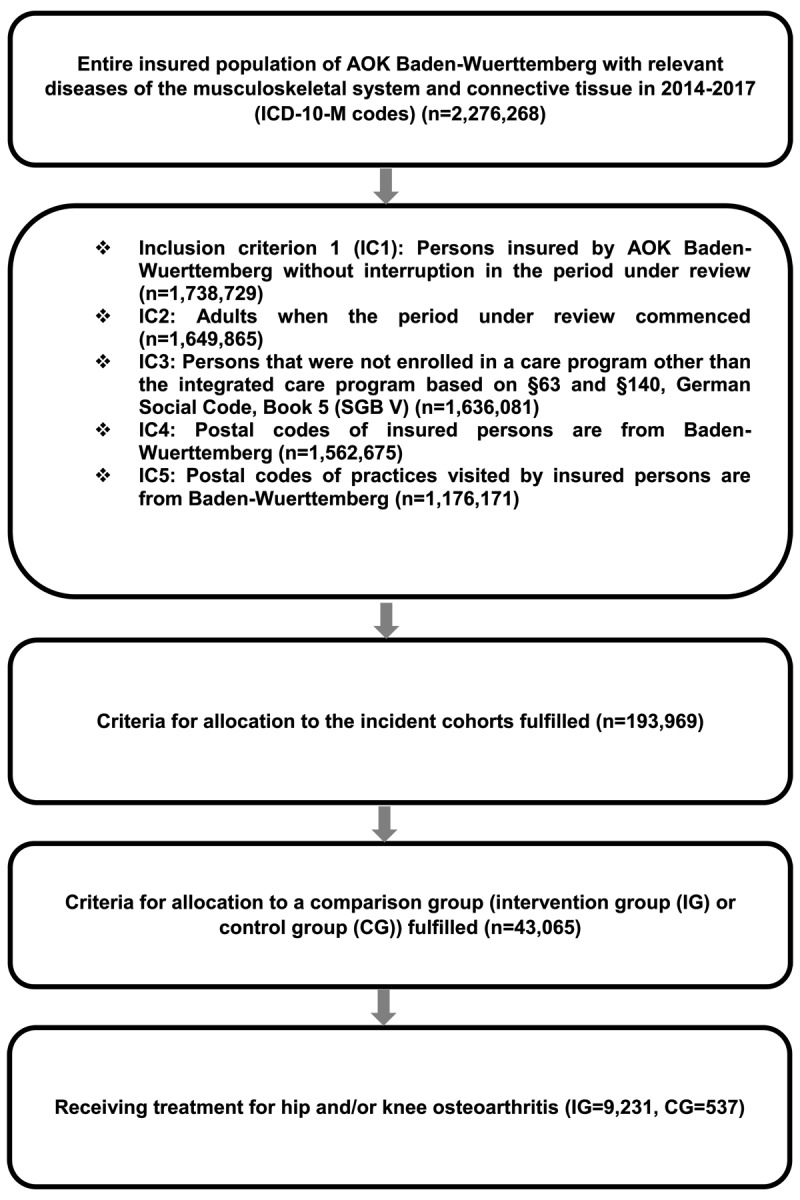
(created by the authors): Prisma diagram for inclusion criteria in study groups.

Patients that did not consult an orthopaedist within 180 days of inclusion, or that switched groups during the observation period, were excluded. A detailed description of the study cohorts and inclusion criteria is shown in Figure 1.

Patients that were enrolled in the programme gave their written informed consent before participation.

This cohort study, which is part of an extensive evaluation report on collaborative care in Germany, was conducted in accordance with the STROBE Statement and the German standard for secondary data analysis (STROSA) [18]. The study has been registered in the German Clinical Trials Register (No. DRKS00017548).

### Orthopaedic care programme

The orthopaedic care programme is a collaborative care model in Baden-Wurttemberg, Germany, for patients with back pain, OA of hip and knee, osteoporosis and rheumatoid arthritis [19]. Details can be found in Book Five of the German Social Code, (SGB V) §73c. Contract partners in the programme are AOK, Bosch BKK (health insurance fund), MEDI-Verbund (Association to promote collaboration between family practitioners and medical specialists), BVOU (Professional Association of orthopaedists and orthopaedic surgeons), BNC (Federal Association of Surgeons), BDRh (Federal Association of German rheumatologists) in collaboration with Rheumaexperten BW eG (an association of practices specialising in rheumatology) and participating orthopaedists.

The aim of the orthopaedic care programme is to provide guideline-oriented care to OA patients in an ambulatory setting. This is achieved by providing financial incentives to promote motivational consultations and patient empowerment, and thus to provide more time for individual consultations. The ultimate aim is to enhance non-pharmacological and non-surgical treatment. Like specialist programmes for other diseases (14), the programme is embedded in the previously described GP-centred healthcare model [13,20]. Participating doctors are encouraged to provide coordinated care, meaning that patients usually see their GP first, who then refers them to a specialist where necessary.

Patients also benefit from high continuity of care, extended consultation times, and lower waiting times for specialist appointments (waiting times are limited to two weeks). Participating orthopaedists are encouraged to perform evidence-based medicine, focus on providing conservative OA therapies, and tailor the information they give to patients to suit their individual needs. This, in turn, requires a thorough assessment of biopsychosocial risk factors and challenges, as well as the appropriate adaptation of therapeutic strategies.

Quality management is also part of the programme and involves doctor participation in regular clinical peer group meetings and continuous data-driven quality improvement. Specifically, doctors are required to participate in at least two structured quality circles per year. The quality circles are organised by the aQua Institute, Göttingen, Germany (Institute for Applied Quality Improvement and Healthcare Research), which prepares information and data on, most importantly, prescription behaviour. This allows physicians to compare their own practice-specific prescription rates to the prescription behaviour of all physicians participating in the orthopaedic care programme [22]. In order to create individual feedback for physicians, aQua also conducts surveys on patient satisfaction and prepares an annual evaluation report focusing on physician-related process evaluation, which describes the indicators agreed upon by the project partners, presents the results of the patient survey over time, and evaluates whether objectives have been reached [22].

Furthermore, doctors involved in a patient’s care communicate seamlessly with one another via a digital communication tool, thus ensuring low rates of information loss.

During the study period, more than 1.6 million patients were enrolled in AOK Baden-Wuerttemberg’s GP-centred healthcare programme [21]. Enrolment is voluntary for both doctors and patients. However, patients have to be enrolled in the GP-centred care programme to participate in the orthopaedic care programme.

### Outcomes

The main focus of the study was on assessing disease-specific hospitalisation. Further health utilisation outcomes were the prescription of physiotherapy and participation in physical exercise programmes. Economic impact was assessed by comparing direct and indirect costs per person per year from a statutory health insurance perspective. Direct costs included those for outpatient visits, statutory treatments (i.e. hospitalisation and rehabilitation), medication and therapeutic remedies and aids. Indirect costs (i.e. productivity losses) were quantified by assessing sick leave payments made by the statutory health insurer to its members. As employers bear the cost of the first six weeks of absence from work, such payments do not generally begin until this period has elapsed. Furthermore, total costs include those for outpatient psychiatric clinics, nursing care, outpatient surgery and patient transport. All information was based on administrative data.

### Statistical analysis

Descriptive statistics were employed to present the data. Differences between groups were tested statistically. We used multivariable analyses based on the covariables shown in Table 1 to deal with imbalances between groups.

**Table 1:** (created by the authors): Baseline characteristics for study groups.


BASELINE CHARACTERISTICS	CONTROL GROUP N = 537	INTERVENTION GROUP N = 9,231

**age (MW[SD])**	67.9[11.7]	64.7 [12.5]

**sex (female)**	65.9%	61.8%

**level of care**¹	2.4%	2.0%

**CCI (MW[SD])**	1.3 [1.6]	1.7 [2.1]

**participation in disease-management programme for type 2 diabetes**	13.0%	20.3%

**cardiovascular comorbidities**	72.4%	69.6%

**type 2 diabetes**	24.4%	25.8%

**stroke and other cerebrovascular diseases**	1.9%	2.6%

**malignoma**	13.6%	15.0%

**obesity**	25.9%	25.5%

**depression**	22.5%	31.0%

**smoking**	3.5%	6.8%

**psychosocial risk factors**	39.1%	41.8%

**burn-out**	6.0%	6.9%

**somatoform disorders**	14.9%	19.2%


¹: Level of care represents the level of impairment for which nursing care is required, 1 being low and 5 high.

**Figure 2:**
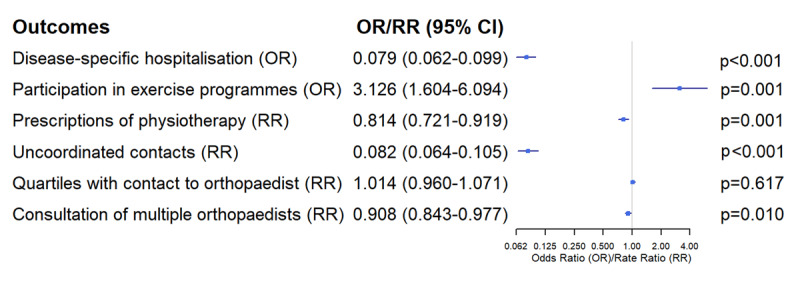
(created by the authors): Individual impact of the covariates on the regression analysis.

Covariables were assessed in the baseline period. The selection of covariables was based on expert knowledge, current literature [23,24,25,26] and availability in the claims data. The Charlson comorbidity index [27] was used to adjust for comorbidities and frailty and any resulting need for nursing care. Generalised linear regression models were used to analyse the intervention effect. The group variable and other covariates (potential confounders) were included in the model as fixed effects. Multivariable analysis was performed to determine effects on health utilisation outcomes, using the covariates (influence factors) listed in Figure 2. Depending on the outcome variable, either logistic or negative-binomial regression models were used. Results were presented as odds ratios (OR) for binary variables and rate ratios (RR) for count variables, with 95% confidence intervals. We considered two-sided p-values and labelled p-values <0.05 as significant.

After using root-mean-square error to compare the predictive properties of different modelling approaches, a linear model was chosen to assess costs. Bootstrapping was used to approximate health care costs for an average AOK Baden-Wuerttemberg cohort. After drawing a random sample (n = 10,000) from the dataset, mean sample costs were estimated by applying the previously calculated model coefficients. The process was repeated until the results stabilised.

We analysed data in accordance with Good Practice in Secondary Data Analysis (GPS) [28]. All descriptive and comparative analyses were carried out using SAS (version 9.4), IBM SPSS Statistics (version 25) and R (version 3.6.2).

## Results

We included 9231 patients in the intervention group and 537 patients in the control group. Mean age of the intervention group was 64.7 ± 12.5 years and of the control group 67.9 ± 11.7 years. The study population was predominantly female in both groups (61.8% vs. 65.9%). The Charlson comorbidity index was higher in the intervention group (1.7 ± 2.1 vs. 1.3 ± 1.6). Depression and other preliminary psychiatric disorders were diagnosed more often in the intervention group (31% vs. 22.5%; 41.8% vs. 39.1%). Further patient characteristics are displayed in Table 1. The results of the regression analysis are listed in Figure 2, along with the individual impact of all covariates.

### Specific hospitalisation

The hospitalisation rate in the intervention group was dramatically lower than in the control group (2.9% vs. 28.7%). The results of the multivariable-adjusted analysis showed that patients in the intervention group were also less likely to be hospitalised for OA (OR 0.079; 95% CI 0.062–0.099; p < 0.0001).

### Exercise programmes and physiotherapy

Participation in physical exercise programmes offered by the AOK was 5.5% in the intervention and 1.7% in the control group. Multivariable analysis showed that patients in the intervention group were more likely to take part in exercise programmes offered by the AOK than patients in the control group (OR 3.126; 95% CI 1.604–6.094; p = 0.001). Overall prescriptions of physiotherapy (including manual therapy, heat therapy and massages) were lower in the intervention group than the control group, 1.8 [SD 2.4] vs. 2.1 [SD 2.8] prescriptions per patient (RR 0.814; 95% CI 0.721–0.919; p = 0.001).

### Collaborative care

Multivariable analysis of the implementation’s success in terms of coordination, continuity and consistency of care, revealed that uncoordinated contacts (e.g. appointments with orthopaedists without prior GP-referral) in the intervention group were significantly lower (RR 0.082; 95% CI 0.064–0.105; p < 0.0001). The number of quartiles (the year consists of four quartiles, with the first extending from January to March) during which orthopaedists were contacted was slightly higher in the intervention group, but not significantly (RR 1.014; 95% CI 0.960–1.071; p = 0.617), and patients were less likely to consult multiple orthopaedists (RR 0.908; 95% CI; 0.843–0.977; p = 0.010).

### Costs

Ambulatory costs attributable to patients in the intervention group (IG) were significantly higher than in the control group (CG) (IG: €1,048; 95% CI €1,042–€1,054; CG: €925; 95% CI €919–€931; Dif.: €123), as were medication costs (IG: €733; 95% CI €722–€743; CG: €678; 95% CI: €667–€688; Dif.: €55). However, the cost of inpatient treatment was €495 lower in the intervention group (IG: €1,003; 95% CI: €986-€1,019; CG: €1,497; 95% CI €1,481–€1,513), which was mainly driven by a significant reduction in hospitalisation costs (IG: €928; 95% CI: €913–€942; CG: €1,300; 95% CI €1,285–1,315; Dif.: €–373). Spending on therapeutic remedies and aids in the intervention group were €11 and €6 lower respectively. Lower productivity was not responsible for any change in indirect costs. Overall, programme participation was associated with significantly lower spending (IG: €3,947; 95% CI €3,893–€4,002; CG: €4,143; 95% CI €4,089–€4,197; Dif.: €–195).

## Discussion

We found the coordinated and continuous care provided to OA patients participating in the collaborative ambulatory orthopaedic care programme to be associated with a reduction in hospitalisation and lower overall costs in patients with emerging OA. Enrolled patients were more likely to participate in exercise programmes and less likely to receive physiotherapy prescriptions. The higher cost of ambulatory care resulted from disease-adjusted reimbursements associated with doctor consultations. Savings from lower hospitalisation rates more than offset higher outpatient costs.

Although lower utilisation of (or spending on) health services is usually considered to be one of the benefits of coordinated care programmes, it should be noted that such an interpretation implies that health care services are overused. However, such an assumption requires the definition of appropriate utilisation and evidence of the delivery of unnecessary care, as there is otherwise a danger that an attempt to prevent overuse may reduce the utilisation of necessary care and have a negative effect on overall healthcare. This study did not assess whether reductions in utilisation or spending were appropriate, but an evaluation of utilisation rates for knee-arthroplasty in OECD countries shows that Germany ranks third in overall utilisation and first in utilisation among patients aged 64 and under. These results suggest that a significant number of surgical procedures are unnecessary [29].

Since consultations conducted as part of the orthopaedic care programme may have beneficial effects on various levels, it is impossible to provide specific explanations for the results. It is possible, however, that doctors use extended consultations to encourage self-management of the disease and that their patients were therefore more likely to exercise and participate in exercise programmes of their own volition, which would be a benefit of conservative therapy. In a meta-analysis comparing the effect of providing advice and information but no further treatment to patients with OA, small but statistically significant improvements were identified in pain and functioning [30]. Similarly, Mazzei et al. concluded in their systematic review that highly structured programmes containing elements of education and exercise are likely to be more cost-effective than usual physician-delivered care in most settings [31].

Our results are consistent with findings from Korean claims data that continuity of care in patients with knee OA reduces hospitalisation costs [26]. Similarly, the results of an Australian study suggest that in patients with chronic diseases, more regular consultations with a GP can lead to a reduction in both cost and the number of days spent in hospital [32]. The observed effects may also reflect the emphasis placed by the orthopaedic care programme on the proactive management of OA, as opposed to reactive and uncoordinated care.

A growing body of evidence highlights the importance of new treatment models in the provision of primary care in OA patients on the basis that they improve patient outcomes and contain costs. These programmes consist of similar elements to the orthopaedic care programme evaluated in this study; i.e. patient consultations, exercise programmes, improved provider coordination, as well as GP training and feedback sessions [33,34,35]. In order to promote the use of clinical practice guidelines in Canadian primary care, “Getting a Grip on Arthritis” [36] introduced a multifaceted integrated client-centred training programme for the management of arthritis in primary care. The project succeeded in enhancing medical care for patients with OA in Canada. Follow-up support, comparable to the clinical peer group sessions in the orthopaedic care programme described in our study, was one of its key features. Other examples in the U.S. show that partial implementation of chronic care models in primary care increase self-efficacy scores in patients with OA [37]. Both the GP-centred healthcare programme and the orthopaedic care programme include elements of stepped-care and chronic care models [13]. Other ideas aimed at substantially improving patient involvement and patient motivation among OA patients in ambulatory care were based on patient-reported outcomes (PROs) and patient-reported outcome measures (PROMs). It is difficult to compare these findings in different patient populations [38] since there is large variation in the use, and a lack of evidence of the usefulness of PROMs both within and between surgical and nonsurgical settings [39]. In the future, novel programmes could profit from using evidence-based PROMs as an essential component for tracking care experience, perhaps supported through the use of digital tools [40]. Such programmes might thus solve the problem that patient activation, although a major component of the programme, has hitherto been largely unmeasurable.

In our study, patients were not likely to receive prescriptions for physiotherapy. However, differing inclusion and exclusion criteria make detailed comparisons with other studies challenging. Some studies recruited patients on the basis of GP referrals of patients with hip or knee OA to the orthopaedic outpatient clinic of a hospital for an orthopaedic consultation to consider hip or knee joint replacement surgery. These studies showed that in addition to usual care, both manual physiotherapy and exercise physiotherapy produce significant improvements in symptoms and physical function in patients with moderate to severe OA of the hips and knees [41]. However, a randomised controlled trial conducted by Bennell at al. found that the 12-week multimodal physical therapy that is currently standard practice in people with symptomatic hip osteoarthritis was no more effective than a realistic sham treatment that controlled for the therapeutic environment, therapist contact time, and home tasks [42].

Our study groups had similar obesity rates (25.9% in control vs. 25.5% in intervention group), with obesity being one of the thoroughly studied risk factors for OA [23,43].

Although participation in exercise programmes was higher in the intervention group, overall participation was very low (4.3%). Exercise is one of the key components of conservative OA therapy [44] and its use should be encouraged. To our knowledge, the orthopaedic care programme reviewed here is unique in that it involves both primary and secondary care and strengthens patient education and motivation.

A comparison of cost data for OA is difficult since patient populations and healthcare resource utilisation are heterogeneous and estimates vary across studies and countries. Differences in data sources and costing lead to further variations in observed costs. In their systematic review, Xie et al. found that annual costs (in US dollars) per patient varied for hospitalisation ($222 to $8,815), outpatient care ($110 to $9,023) and medication ($12 to $3,624). Total annual direct costs ranged from $1,442 to $21,335. A German study found that medication and non-physician services provided to OA patients cost €699 and €171 respectively [45]. Assuming the latter category is similar to what we have referred to as therapeutic remedies, these figures are comparable to those in our control group. In the same study, outpatient consultations, inpatient treatment and direct costs were estimated to be €357, €175, and €1,511 respectively [45]. These figures are much lower than those observed in our study, possibly reflecting differences in methodological approaches. While our study used statutory health insurance claims data, Sabariego et al. obtained cost estimates retrospectively via a self-reported questionnaire. Previous research suggests that direct costs taken from claims data are generally higher than those based on survey data [5].

The strengths of our study are the large sample size and the real-world population-based approach, as the group of patients was large enough to permit advanced statistical modelling, and real-world data was available for multiple health care sectors. The use of claims data further enabled us to eliminate recall bias but simultaneously to rely on coding quality. A major limitation of the study is that the methodology and use of claims data means we have no laboratory data and medical records, and no details on level of pain and functional impairment. Additionally, consultations may have varied between groups. Furthermore, patient knowledge was not assessed at any time during the study. Our choice of selected outcomes reflects the scarcity of evidence-based and valid quality indicators for the assessment of osteoarthritis in primary care [46]. However, the outcomes have also been used in previous evaluations. As minor symptoms are likely to remain undiagnosed and referrals to orthopaedists occurred within 180 days of the original diagnosis, it can be assumed that our study cohort first consulted a GP with symptoms and/or clinical evidence.

We adjusted for a wide range of covariates. Nonetheless, residual confounding from unmeasured confounders cannot be ruled out. Since patients and doctors participate in the programme voluntarily, self-selection bias undoubtedly plays a role in both GP-centred and orthopaedic care programmes. Furthermore, to avoid group contamination we excluded patients that were enrolled in GP-centred care but did not consult a specialist participating in the programme.

## Conclusion

Collaborative ambulatory orthopaedic care is a novel healthcare programme that exploits options for the conservative treatment of OA outpatients in an attempt to reduce the likelihood of hospitalisation. Our results show that despite increased ambulatory care spending, overall annual cost savings amounted to €195 per patient in individuals with hip and/or knee OA. Considering even a moderate OA prevalence rate of 15.3% for Germany, this implies the broad implementation of such a programme may lead to substantial cost savings.
